# Quality of intrapartum care at Mulago national referral hospital, Uganda: clients’ perspective

**DOI:** 10.1186/1471-2393-13-162

**Published:** 2013-08-13

**Authors:** Omar Kigenyi, Getachew B Tefera, Elizabeth Nabiwemba, Christopher G Orach

**Affiliations:** 1 School of Public Health, College of Health Sciences, Makerere University, Kampala, Uganda; 2 Regional Center for Quality of Health Care, School of Public Health, College of Health Sciences, Makerere University, Kampala, Uganda; 3 Department of Community Health and Behavioral Sciences, School of Public Health, College of Health Sciences, Makerere University, Kampala, Uganda

**Keywords:** Quality of care indicators, Client centered care, Clients’ perspective, Intrapartum care, Maternity care services

## Abstract

**Background:**

Quality of intrapartum care is an important intervention towards increasing clients’ utilization of skilled attendance at birth and accelerating improvements in newborn’s and maternal survival and wellbeing. Ensuring quality of care is one of the key challenges facing maternal and neonatal services in Uganda. The study assessed quality of intrapartum care services in the general labor ward of the Mulago national referral and teaching hospital in Uganda from clients’ perspective.

**Methods:**

A cross sectional study was conducted using face to face interviews at discharge with 384 systematically selected clients, who delivered in general labor ward at Mulago hospital during May, 2012. Data analysis was done using STATA Version (10) software. Means and median general index scores for quality of intrapartum care services were calculated. Linear regression models were used to determine factors associated with quality of care.

**Results:**

Overall, quality of intrapartum care mean index score was 49.4 (standard deviation (sd) 15.46, and the median (interquartile range (IQR)) was 49.1 (37.5–58.9). Median index scores (IQR) per selected quality of care indicators were; dignity and respect 75 (50–87.5); relief of pain and suffering 71.4 (42.8-85.7); information 42.1 (31.6-55.3); privacy and confidentiality 33.3 (1–66.7); and involvement in decision making 16.7 (1–33.3). On average, higher educational level (college/university) (β: 6.81, 95% CI: 0.85-15.46) and rural residence of clients (β: 5.67, 95% CI: 0.95-10.3) were statistically associated with higher quality scores.

**Conclusion:**

This study has revealed that quality of intrapartum care services from clients’ perspective was low. Improvements should be focused on involving clients in decision making, provision of information about their conditions and care, and provision of privacy and confidentiality. There is also need to improve the number and availability of health care providers in the labor ward.

## Background

The intrapartum and immediate postnatal periods [1] are a time of significant risk to both mother and her child [2]. Adverse intrapartum events are implicated in 23% of neonatal deaths, 32% of stillbirths and 42% of maternal deaths as well as a poorly measured burden of long-term impairment and disability worldwide [3]. In developing countries particularly, professional care is beyond the reach of many women [4]. Sub-Saharan Africa alone approximates about 77% of neonatal deaths and a similar proportion of maternal deaths [5].

In Uganda, most maternal deaths occur during delivery and during the postpartum period. Many women suffer injuries, infections, and disabilities brought about by pregnancy or childbirth complications [6]. On a daily basis, 16 women die during childbirth [7]. For every woman who dies, six survive with chronic and debilitating ill health [8]. Direct causes of maternal deaths during the period of giving births are haemorrhage (34%), sepsis/infections (10%), hypertensive disorders (9%) and ectopic pregnancy (5%) [9].

Key maternal mortality reduction strategies including family planning, skilled care attendance during antenatal, delivery and postpartum period, emergency obstetric and newborn care have been encouraged in developing countries. In Uganda, the safe motherhood initiative was introduced in the early 1990s with the aim of halving maternal mortality by the year 2000. This objective was not achieved. In 2003, the proportion of established posts filled by qualified health workers was increased from 34% in 1999 to 53% [10], and to 68% in 2004/05 [8]. In the 2004 through the poverty eradication action plan, the government reaffirmed its commitment to achieve the millennium development goals [8]. The Uganda national minimum health care package was introduced during health sector strategic plan (HSSP) 1, through HSSP II, and HSSP III, where maternal and child health were among the key focus areas. In 2007 and 2009, the road map to accelerate reduction of maternal and neonatal morbidity and mortality, and the national child survival strategy were formulated [11]. In the September, 2011 UN General Assembly in New York, Uganda re-committed to implement the Global strategy for women's and children's health [7].

Maternal and neonatal indicators remain poor in Uganda. Institutional delivery under skilled care is 57%, maternal mortality ratio is 438/100,000 live births, and neonatal mortality rate is 27/1000 live births. Ensuring quality of care is a key challenge facing maternal and neonatal services in Uganda. Quality of care is observed when the care provided is safe, effective and takes account of the patients’ experience [12,13]. Measures of quality focus on the structure, process and outcomes [14]. However, little attention has been paid to evaluating the quality and practices of care [4] in public or private hospital settings [15].

Systematic assessment of quality and practices of care during labor focusing on data collected from clients can be used to document and improve quality of care at the health facility level. The evaluation of quality is important in deciding which aspects of care need to be improved.

In line with the road map to accelerate reduction of maternal and neonatal morbidity and mortality of the MOH (2006) vision, to have women in Uganda go through pregnancy, childbirth and postpartum periods safely and their babies born alive and healthy, and the goal, to accelerate the reduction of maternal and neonatal morbidity and mortality in Uganda.

The study assessed the degree to which the health care system effectively renders care on an individual level during labor at the labor ward. It aimed at generating information and identifying gaps in intrapartum care using clients’ experiences with services so as to inform hospital managers, clinicians, and policy makers about where improvements may be focused. The objectives of the study were to establish client’s perspective on quality of intrapartum care services in the general labor ward and to determine factors associated with quality of care.

## Methods

A cross sectional study was conducted using face to face interviews. Data were collected in May 2012 at the general labor ward in the Mulago hospital. Mulago hospital is the national referral and teaching hospital for Makerere University College of Health Sciences. It is the largest hospital and oldest training institution for most physicians and other health professionals in Uganda. On average Mulago attends to 700,000 outpatients, and 140,000 inpatients per year [16]. Being a national referral hospital, the general labor ward receives normal deliveries as well as high risk mothers with maternal or obstetric complications referred at various stages of pregnancy from all over the country.

Kish-Leslie formula (1965), was used to determine the sample size of 384 with assumptions that Z^2^ test statistic at 95% CI = 1.96, prevalence for the outcome 50% (no prior published studies in this or a similar setting were found), Q at 100%-P and precision = 5%.

The study participants were women who delivered from the Mulago hospital general labor ward, who were in good health condition and at discharge. Women with severe post delivery complications (for example severe bleeding, ruptured uterus, hypertensive disorders, and psychiatric diagnosis were excluded).

The participants interviewed were selected using systematic sampling technique. The first respondent was randomly obtained using the fish bowl method of selection. On average, 20 respondents met the selection criteria and were daily recruited to participate in the survey. Informed consent was obtained from respondents prior to the interview.

### Quality control

Survey questionnaire was pre-tested to ensure the validity and reliability of the tool. Survey questions included 26 items in line with client centered care indicators. The items were constructed using likert scale (with categories ranging from ‘never to always’), or a nominal scale (with categories of ‘no and yes’). Responses from a likert scale were recorded on a three & four point scale while responses from nominal scale were recorded as one and two. Higher scores of responses reflected higher quality for both likert and nominal scales.

### Variables and measurements

The primary outcome was quality of intrapartum care services. It was measured as a continuous variable constructed as a composite variable from the total of 26 question items including index scores of quality of care indicators from dignity and respect [three items], relief of pain and suffering [three items], health promotion [seven items], information [eight items], privacy and confidentiality [one items], satisfaction with waiting time [one items], priority to sick individuals [one items], and involvement in shared decision making [two items]. Quality of intrapartum care was defined as a binary variable “low” and “high” on a continuous scale 0 to 100. A mean score less than 50% was termed as low and a mean score greater than or equal to 50% termed as high.

Independent variables expected to influence client’s perspective of quality of care were summarized as clients’ factors, and were measured by total number of children ever born, age in complete years, marital status, occupation, education level, place of residence, and referral.

### Analyses

Raw data were captured using Epidata 3.0 software. Data were cleaned, edited to identify out of range values, missing values or any other inconsistencies. Data were exported to STATA version (10) software for analysis.

The scores of quality of intrapartum care services constructed as continuous composite variable(s) were then transformed into a 0 to 100 scale using Sloan formula, below.

$$Y=[100*[\left(\mathrm{RS}-\mathrm{MIN}\right)/\left(\left(\mathrm{MAX}-\mathrm{MIN}\right)\right]$$

Where MIN = minimum possible raw scale value if all items were answered, MAX = maximum possible raw scale value if all items were answered, RS = participant’s raw score for quality of intrapartum care services or each quality of care indicator, and Y = participant’s transformed score for quality of intrapartum care services or each quality of care indicator.

This transformation facilitated interpretation and comparison between indicators and studies given that the primary outcome was continuous [17].

Means and median index scores of quality of intrapartum care services or quality of care indicators were then obtained. A mean/median score less than 50% was termed as low and a mean/median score greater than or equal to 50% termed as high.

Data from quality of care indicators were skewed. The median was the best measure that would produce unbiased estimates compared to the mean. This helped to avoid spurious conclusions. To allow detailed comparison and ready interpretations, the coefficient of skewness based on the quartiles was determined. This measure, unlike the coefficient of skewness based on the third moment, does not disproportionately inflate with the presence of a single unusually large or unusually small value. The coefficient of skewness indicates the degree and direction of skew.

Definition of coefficient of skewness based on quartiles [18].

$$Sk_{4}=\left[\left(Q_{3}-M\right)-\left(M-Q_{1}\right)\right]/\left(Q_{3}-Q_{1}\right)$$

Where Sk4 = coefficient of skewness, Q_1_ = quartile 1 score, Q_3_ = quartile 3 score and M = median score.

When making interpretations, if the direction of skewness Sk = 0: symmetric, Sk > 0: positively skewed, Sk < 0: negatively skewed. The farther skew is from 0, the more skewed the distribution. If the distribution is symmetric then the distance between Q_1_ and the median must be the same as the distance between the median and Q_3_[18].

Linear regression models were used to obtain expected bi-variate and multi-variate quality of care scores, 95% confidence intervals and p-values for client’s characteristics that affect quality of care.

Beta coefficients from the general linear models, for all independent variables were shown. Positive values of a variable category indicated higher perceived quality (satisfaction) relative to the referent category level. Negative values indicated a decreasing perceived quality compared to the reference category. The level of statistical significant was at p-valve ≤ 0.05.

### Ethical approval

The study protocols were approved by Makerere School of Public Health Higher Degrees Research and Ethics Committee on 22nd March 2012 and the Mulago Hospital Research and Ethics Committee, MREC no. 191 of 11th April 2012.

## Results

During May, 2012, 384 clients participated in the survey. Their mean age was 24.1 years (SD, 5.04), median of 23 years and a range of 18 to 41 years. Majority 333 (87%) lived in urban setting and 176 (46%) had attained secondary level education.

Most participants 159 (41%) had one child and 141 (37%) had between 2 to 3 children ever born. More than half of clients 206 (54%) came to the hospital directly without referral. Baseline characteristics of study participants are shown in Table 1.

**Table 1:** Overall scores for quality of care indicators by client’s characteristics at Mulago hospital, general labor ward during May, 2012

**Characteristics**	**Frequency n (%)**	**Mean index scores (SD)**	**Median index scores (IQR)**	**P-value (difference in mean scores)**
**Overall**	384	49.4 (15.46)	49.1 (37.5-58.9)	
**Age group (years)**				0.5
18–19	86 (22.4)	50.4 (17.8)	50 (37.5 - 62.5)	
20–24	141 (36.7)	49.7 (14.4)	49.1 (39.3 - 58.9)	
25–29	85 (22.1)	47.1 (15.4)	48.2 (37.5-53.6)	
≥ 30	72 (18.6)	47.0 (21.5)	48.2 (25–67.8)	
**Educational level**				
No education	21 (5.5)	44.9 (13)	50 (37.5-46.4)	0.0111*
Primary	134 (34.9)	46.6 (15.2)	44.6 (35.7-58.9)	
Secondary	176 (45.8)	51.0 (15.4)	51.8 (39.3-59.8)	
Post secondary (college/university)	53 (13.8)	53.0 (16.0)	55.4 (41.1-60.7)	
**Current occupation**				
House work	217 (56.5)	49.1 (16)	48.2 (37.5-58.9)	0.8
Employed/salaried	62 (16.2)	49.7 (15.3)	50.9 (41.1-58.9)	
Self employed/business	105 (27.3)	50 (14.4)	50 (39.3-60.7)	
**Current marital status**				
Never married	64 (16.7)	48.6 (15.7)	48.2 (37.5-58.9)	0.8
Currently married	320 (83.3)	49.1 (15.6)	46.4 (39.3-57.1)	
**Total number of children ever born**				
1	159 (41.4)	51.6 (16.1)	51.8 (39.3- 62.5)	0.05*
2–3	141 (36.7)	48.5 (15.4)	46.4 (37.5-58.9)	
4–5	56 (14.6)	45.4 (13)	43.7 (37.5-53.6)	
6+	28 (7.3)	49.8 (15.2)	50 (42.8-60.7)	
**Place of residence**				
Urban	333 (86.7)	48.7 (15.34	48.2 (37.5- 58.9)	0.03*
Rural	51 (13.3)	53.67 (15.69)	53.6 (42.8- 62.5)	
**Referral**				
No	206 (53.6)	49.6 (15.9)	50 (37.5-58.9)	0.8
Yes	178 (46.4)	49.2 (14.9)	48.2 (39.3-58.9)	

**Key:** mean age of respondents: 24.1 years (SD: 5.04), median age: 23 years, range: 18 to 41 years.

*statistically significant at ≤ 0.05

### Quality of intrapartum care services

#### Socio-demographic factors that affect the clients’ perspective of quality

Overall clients’ mean index score was 49.4 (sd) 15.46. Bi-variate analyses indicate that there were no statistically significant differences in overall scores of quality of care indicators by age group (p = 0.5), current occupation, marital status, and referral (p = 0.8) respectively. There were statistically significant differences in the clients’ overall scores for quality of care indicators by education level (p = 0.01), number of children ever born (p = 0.05), and place of residence (p = 0.03). These are shown in Table 1.

In the multivariate model, socio-demographic factors statistically associated with overall mean quality of care indicators were education level, place of residence, and number of children ever born. On average, quality of care was significantly higher for post secondary women relative to those with no education (β: 6.81, 95% CI: 0.85-15.46) and rural residents relative to urban residents (β: 5.67, 95% CI: 0.95-10.3). However, quality of care was significantly lower for women with 4–5 children ever born relative to women with 1 child (β: -4.04, 95% CI:-3.65--0.03) as outlined in Table 2.

**Table 2:** Linear regression coefficients of overall mean of quality of care indicators by clients’ characteristics

**Variable**	**Bi-variate analyses**		**Multi-variate analyses**		**P-value**
	**β 95% CI**		**β 95% CI**		
**Age group (years)**					
18–19 (reference)					
20–24	−0.67	−4.53 3.18	−0.64	−5.35 4.07	0.7
25–29	−3.33	−8.25 1.58	−3.31	−10.36 3.73	0.4
≥ 30	−3.41	−21.28 14.46	−7.32	−26.9 12.27	0.5
**Educational level**					
No education (reference)					
Primary	1.59	−5.46 8.65	0.62	−6.75 7.98	0.8
Secondary	6.03	−0.91 12.97	5.27	−2.15 12.69	0.2
Post secondary (college/university)	8.05	0.29 15.80*	6.81	0.85 15.46	0.023*
**Current occupation**					
Housework (reference)					
Employed/salaried	0.63	−3.76 5.02	−0.58	−5.31 4.15	0.8
Self employed/business	0.95	−2.67 4.57	1.72	−2.15 5.59	0.4
**Current marital status**					
Never married (reference)					
Currently married	0.51	−4.53 5.55	−0.22	−3.15 5.09	0.9
**Total number of children ever born**					
1 (reference)					
2–3	−3.13	−6.62 0.37	−2.67	−1.98 0.46	0.2
4–5	−6.14	−10.8 -1.45*	−4.04	−3.65 -0.03	0.04*
6+	−1.71	−7.9 4.48	1.58	−3.46 1.82	0.7
**Place of residence**					
Urban (reference)					
Rural	4.92	0.36 9.46*	5.67	0.95 10.38	0.019*
**Referral**					
No (reference)					
Yes	-.38	−3.49 2.72	−0.01	−3.16 3.14	0.9

**Key:** β, Beta coefficients are the regression parameters from the overall linear models (bi-variate and multi-variate analyses). Positive values indicate higher quality relative to the referent variable category level, while negative values indicate lower quality compared with the referent category.

*statistically significant at p-valve ≤ 0.05, 95% CI excludes a zero (0).

Table 3 shows general scores on the main indicators of quality of care from the client’s perspective. Results are explained per main indicator below.

**Table 3:** General scores of quality of intrapartum care services according to quality of care indicators at the Mulago hospital general labor ward during May, 2012

**Quality of care indicators**	**Range of scores**	**Median index scores**	**Quartile 1 (Q**_**1**_**)**	**Quartile 3 (Q**_**3**_**)**	**Coefficient of skewness (based on Quartiles)**
Dignity and respect	12.5–100	75	50	87.5	- 0.33
Relief of pain and suffering	0-100	71.4	42.8	85.7	−0.03
Health promotion	0-100	42.85	14.3	71.4	0
Information	5.3-100	42.1	31.6	55.3	0.11
Satisfaction with waiting time	0–100	33.3	1	66.7	0. 02
Privacy and confidentiality	0-100	33.3	1	66.7	0.02
Priority to sick individuals	0-100	33.3	1	66.7	0.02
Involvement in decision making	0-100	16.7	1	33.3	0.03

**Key:** Negative skewness means that most values were clustered at the right end of the median so that the median is closer to Q_3_ than Q_1_. Positive skewness means that most values clustered at the left end of the median as a result, the median is closer to Q_1_ than Q_3_. Zero means symmetric

The general median index score (IQR) for dignity and respect was 75 (50–87.5), and for relief of pain and suffering was 71.4 (42.8-85.7). More than half of clients had index score between 75% and 87% for dignity and respect and index score between 71% and 86% for relief of pain and suffering.

The general median index score, (IQR) for satisfaction with waiting time was 33.3 (1–66.7), for privacy and confidentiality was 33.3 (1.-66.7), and for involvement in decision making was 16.7 (1–33.3). More than half of clients had index score between 1% and 33.3% for satisfaction with waiting time and for privacy and confidentiality. Over three quarters of clients had index score between 1% and 17% for involvement in decision making.

#### Health promotion

The general median index score (IQR) for health promotion was 42.8 (14.3-71.4). Further analysis as presented in Table 4, indicates that the percentage of clients who received education on selected health promotion activities were personal hygiene 214 (56%); immunization 208 (54%); breast feeding 201 (52%); diet 183 (48%), family planning 153 (40%), ambulation 145 (38%), and cancer screening 77(20%).

**Table 4:** Percent of clients provided health promotion education and information

**Type of education/variable**	**Frequency (n = 384)**	**Percent (%)**
**Clients educated on health promotion**		
Personal hygiene	214	56
Immunization	208	54
Breast feeding	201	52
Diet	183	48
Family planning	153	40
Ambulation	145	38
Cancer screening (breast and cervical cancer)	77	20
**Clients provided with information**		
Direction to service deliver site	283	74
Communicated to, in language understood	249	65
Listened to whenever with concern	234	61
Given information on expected/emerging symptoms or health problems after delivery	145	38
Told reasons before performing any procedure or treatment	42	11
Given opportunity for questions and discussions	39	10
Obtained information on their care preferences	34	9
Explained any possible side effect, before any procedure or treatment	31	8

#### Information

The general median index score (IQR) for information was 42.1 (31.6-55.3). Table 4 indicates detailed analysis of information. It reveals that 234 (61%) of clients were listened to whenever they had a concern, 38% received information on expected symptoms or health problems after delivery. And less than 15% of clients were told reasons for performing any procedure or treatment, were explained possible side effect, or given opportunity to ask questions.

## Discussion

This was a cross sectional study conducted to assess quality of intrapartum care by state services provided in the Mulago national referral hospital in Uganda. The participants were women who delivered in the general labor ward and discharged in May 2012. The study revealed that quality of intrapartum care services from the perspective of clients was low. The overall mean index of quality of care services was 49.4%.

The findings of the study are consistent with the earlier studies conducted on quality and satisfaction with services. A study conducted at the Mulago hospital in outpatient clinics revealed that patient overall satisfaction with services was suboptimal, (mean score: 2.7) on a scale of (1 to 4) [19]. In the survey by Maternal and Neonatal Program Effort Index (MNPI) conducted in Uganda, the average rating for delivery care services were at 55 out of 100. Uganda’s ratings for maternal health services were lower than the global average in some key areas [6].

In the current study, factors that influenced rating of quality of maternity and newborns care services during labor were several. Information flow between clients and medical staff was an important factor identified in our study as influencing quality of care. The percentage of clients given opportunity to ask questions or discussions in our study was 10%, clients whose information on care preferences obtained was 9%. This may explain why involvement in decision making had the least median index score of about 17% and information sharing with median index score of 42%. Possible explanation to this finding is the small number of medical staff in relation to large daily client load at the labor ward. As a result, this affects the client-provider interactions, health services delivered, health status of clients, and clients’ satisfaction. Thus influencing clients’ perspective on quality of care and eventually leading to sub-optimal quality for intrapartum care services in the hospital.

Findings from a study conducted in the Gambia about quality of intrapartum care identified failure of health workers to inform women about procedures and involving clients in their own health care as a common practice [4].

In other studies, it is shown that the system or health care providers may fail their customers. Most of the failures include disrespect, inconvenience, poor communication, and fragmentation [20]. Staff have poor attitude due to unfair reward system [16]. Providers in Uganda have poor attitudes and lack skills needed for individual interactions as well as skills for working with larger community [11]. Other studies from developing countries have also reported that when staff numbers and staff morale is low, it may be cited as a reason for poor provision of services [21-23], as a result quality of care experienced by women in labor become poor [24].

Improvements in quality of care need to address both human aspects of care and health systems issues, such as supply of key drugs, as well as changes in professional practice [25].

Specifically, some studies have suggested that quality in maternity care requires mothers to be involved in decision making and being seen as partners in health care [26]. A qualitative study conducted among health care consumers identified aspects of the hospital experience most important to improved health outcome and client centered care in maternity care. These were respect for patients’ values; preferences and expressed needs; coordination and integration of care; information, communication and education; physical comfort; emotional support; involvement of family and friends; transition and continuity; and access to care [27].

Providers should have the ability to listen actively, engage mothers, demonstrate cultural sensitivity and give support to clients [28]. The directorate of obstetrics and gynecology in Mulago hospital is working towards achieving its mission of providing state of the art reproductive health care [16].

### Strengths and limitations

The study contributes to quality improvement programs responsible for accelerating reduction of maternal and neonatal morbidity and mortality in Uganda. It documents and informs clinicians, hospital managers, and policy makers about quality of care aspects that need to be improved in promoting newborns and maternal survival and wellbeing during labor. It also provides information necessary to promote women’s utilization of skilled attendance at birth. To our knowledge, this is one of the first systematic assessments of quality of intrapartum care services at the general labor ward in a national referral hospital in Uganda.

This is a cross sectional study which gives a snapshot of events, and the study was conducted without a comparative group. Quality is a process of improvement and depends upon several factors such as type and availability of staff, drugs, technical and interpersonal relationships with clients and amongst providers, from time to time. We were not able to undertake observation as a verification method for the data given since the study was based on interviews with clients about their experiences with intrapartum care services received. We were also unable to obtain information from women with severe post delivery complications (severe bleeding, and ruptured uterus). These severe complications could have been probably explained by the care rendered to these clients.

## Conclusion

The study has revealed that quality of intrapartum care services from the perspective of clients was low. Quality of care indicators had low scores pertaining to the way care was being delivered appropriately, skillfully and in a human manner consistent with client’s preferences and culture by the medical staff throughout the course of labor and delivery at the labor ward.

These results suggest that more should be done to improve quality of maternity care services offered at the labor ward. Improvements should be focused on involving clients in decision making, information about their conditions and care, and provision of privacy and confidentiality. There is also need to improve availability of health care providers and conducting regular client quality assessment surveys to identify aspects of care that need improvement in meeting clients’ expectations for services.

### Further research

Because quality improvement focus on the client, systems and processes, teamwork, and the use of data, there is need to further understand broader organizational factors and inter-professional relationships. Contextualizing the providers’ perspective, can give a comprehensive understanding of quality improvement opportunities at the general labor ward of the Mulago national referral hospital.

## Competing interests

The authors declare that they have no competing interests.

## Authors’ contributions

OK: planned the study, developed the methodology, questionnaire, and participated in data collection and entry. Planned, conducted the analysis, and drafted the manuscript. GBT: supervised the planning of the study, development of the methodology, questionnaire, analysis plan, and manuscript writing. EN: helped in writing the manuscript. CGO: supervised the planning of the study, development of the methodology, questionnaire, analysis plan, and manuscript writing. All authors read and approved the final manuscript.

## Pre-publication history

The pre-publication history for this paper can be accessed here:

http://www.biomedcentral.com/1471-2393/13/162/prepub
